# Errata

**Published:** 1888-08

**Authors:** 


Errata.—In the very interesting report of a case of Retinitis Pigmentosa, given in our June issue, by Dr. Fredk. W. Payne, of Boston, *Arum* was written for *Aurum* (see p. 290, lines 6 and 10). In the lecture upon Podophyllum, p, 282, line 18, read *Prolapsus of rectum when urinating, Mur-ac.* In the text *vomiting* was given for *urinating.* In our May issue, p. 243, line 12, from bottom, Dr. Biegler is reported as giving *ten* remedies in a case where *two* cured.

